# 2,2′-(4-{[(*E*)-4-Meth­oxy­benzyl­idene]amino}­phenyl­imino)­diethanol

**DOI:** 10.1107/S1600536810028035

**Published:** 2010-07-31

**Authors:** Xiaoju Liu, Bingqin Yang, Mingjuan Wang, Maxim V. Borzov

**Affiliations:** a Key Laboratory of Synthetic and Natural Chemistry of the Ministry of Education, College of Chemistry and Material Science, The North-West University of Xi’an, Taibai Bei Avenue 229, Xi’an 710069, Shaanxi Province, People’s Republic of China

## Abstract

In the title compound, C_18_H_22_N_2_O_3_, the dihedral angle between the aromatic rings is 3.9 (2)°. Both H atoms of the hy­droxy groups are involved in inter­molecular O—H⋯O hydrogen bonding. In the crystal structure, this hydrogen bonding assembles mol­ecules into chains of 2_1_ symmetry extending parallel to the *b* axis. The almost planar (within 0.09 and 0.06 Å) 4-CH_3_O–C_6_H_4_–CH=N–C_6_H_4_– groups are oriented outwards the twofold screw axis.

## Related literature

For practical inter­est in Shiff bases of general type *p*-*R*′–C_6_H_4_–CH=N–C_6_H_4_–*R*′′-*p* in various areas, see: von König *et al.* (1982 ▶); Haldavanekar *et al.* (2009 ▶); Ferlin *et al.* (2004 ▶); Lewis *et al.* (2009 ▶). For the only two structurally characterized compounds of this type with *R*′′ = N(alk­yl)_2_, see: Nagao *et al.* (2002 ▶); Nakai *et al.* (1976 ▶). For 4-[(*E*)-({4-[bis­(2-hy­droxy­eth­yl)amino]­phen­yl}imino)­meth­yl]phenol, C_17_H_20_N_2_O_3_, see: Liu *et al.* (2010 ▶). For a description of preparation routines, see: Cho & Park (1997 ▶); Ferlin *et al.* (2004 ▶); von König *et al.* (1982 ▶). For a description of the Cambridge Structural Database, see: Allen (2002 ▶).
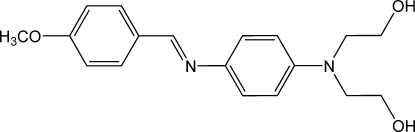


## Experimental

### 

#### Crystal data

C_18_H_22_N_2_O_3_
*M* *_r_* = 314.38
Monoclinic, 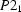

*a* = 5.3795 (9) Å
*b* = 8.0585 (14) Å
*c* = 18.531 (3) Å
β = 91.168 (2)°
*V* = 803.2 (2) Å^3^
*Z* = 2
Mo *K*α radiation
μ = 0.09 mm^−1^
*T* = 296 K
0.24 × 0.13 × 0.07 mm

#### Data collection

Bruker SMART APEXII diffractometer
Absorption correction: multi-scan (*TWINABS*; Sheldrick, 1996 ▶) *T*_min_ = 0.979, *T*_max_ = 0.994
4036 measured reflections
1541 independent reflections
1225 reflections with *I* > 2σ(*I*)
*R*_int_ = 0.037

#### Refinement

*R*[*F* ^2^ > 2σ(*F* ^2^)] = 0.038
*wR*(*F* ^2^) = 0.108
*S* = 1.06
1541 reflections
222 parameters
1 restraint
H atoms treated by a mixture of independent and constrained refinement
Δρ_max_ = 0.17 e Å^−3^
Δρ_min_ = −0.13 e Å^−3^

### 

Data collection: *APEX2* (Bruker, 2007 ▶); cell refinement: *SAINT* (Bruker, 2007 ▶); data reduction: *SAINT*; program(s) used to solve structure: *SHELXS97* (Sheldrick, 2008 ▶); program(s) used to refine structure: *SHELXL97* (Sheldrick, 2008 ▶); molecular graphics: *SHELXTL* (Sheldrick, 2008 ▶) and *OLEX2* (Dolomanov *et al.*, 2009 ▶); software used to prepare material for publication: *SHELXTL* and *OLEX2*.

## Supplementary Material

Crystal structure: contains datablocks I, global. DOI: 10.1107/S1600536810028035/im2204sup1.cif

Structure factors: contains datablocks I. DOI: 10.1107/S1600536810028035/im2204Isup2.hkl

Additional supplementary materials:  crystallographic information; 3D view; checkCIF report

## Figures and Tables

**Table 1:** Hydrogen-bond geometry (Å, °)

*D*—H⋯*A*	*D*—H	H⋯*A*	*D*⋯*A*	*D*—H⋯*A*
O2—H2⋯O3^i^	0.95 (5)	1.84 (6)	2.762 (3)	177 (4)
O3—H3⋯O2^ii^	0.82 (4)	1.95 (4)	2.757 (4)	166 (4)

Symmetry codes: (i) 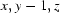; (ii) 
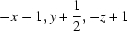
.

**Fig. 1.**
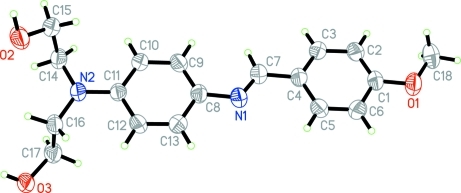
Asymmetric unit of (I) showing the labelling scheme and thermal ellipsoids at the 50% probability level.

**Fig. 2.**
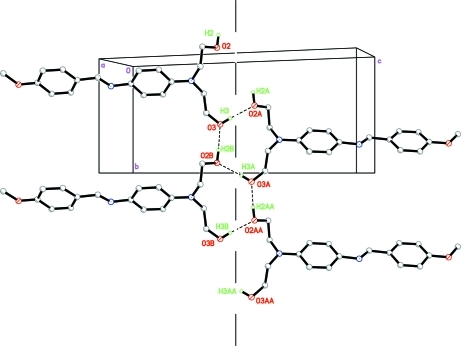
View of a 21-symmetrical chain assembled by molecules of (I) via hydrogen bonds. Hydrogen atoms except of the OH ones are omitted for clarity. Labelling is provided only for atoms involved in H-bonding. H-bonds are depicted as dashed lines. 2-fold screw axis is depicted as a long-dash line.

## supplementary crystallographic information

### Comment

Shiff bases of the general type *p*-R'–C_6_H_4_–CH=N–C_6_H_4_–R"-*p* are well-known compounds that find practical application in various areas [photography (for instance, see von König *et al.*, 1982), medicinal and pharmaceutical chemistry (for instance, see Haldavanekar *et al.*, 2009; Ferlin *et al.*, 2004; Lewis *et al.*, 2009)]. Recently, we were interested in preparation of a series of 2-((2-hydroxy-ethyl)-{4-[(benzylidene)-amino]-phenyl}-amino)-ethanols as intermediates for their further conversion into paracyclophanes. This way, 2-((2-hydroxy-ethyl)-{4-[(*(1E)*4-methoxy-benzylidene)-amino]-phenyl}-amino)-ethanol , C_18_H_22_N_2_O_3_, (I), and 4-({*(E)*-4-[bis-(2-hydroxy-ethyl)-amino]-phenylimino}-methyl)-phenol , C_17_H_20_N_2_O_3_, [II; Liu *et al.* (2010)] were prepared by a condensation reaction between 2-[(4-Amino-phenyl)-(2-hydroxy-ethyl)-amino]-ethanol and 4-methoxy- or 4-hydroxybenzaldehyde, respectively.

Despite of the fact that structurally characterized Shiff bases of general type *p*-R'–C_6_H_4_–CH=N–C_6_H_4_–R"-*p* are well presented in the Cambridge Structural Database [CSD; Version 5.27, release February 2009; Allen, 2002; 128 entries, 173 fragments], among them there are only two compounds with R" = *N*(alkyl)_2_ [namely: R' = H, R'' = NEt_2_ (Nagao *et al.*, 2002) and R' = NO_2_, R" = NMe_2_ (Nakai *et al.*, 1976)]. From this viewpoint, X-ray single crystal study of (I) presents a certain descriptive interest.

The asymmetric unit of (I) is shown in Fig. 1. Except of dihedral angle C7–N1–C8–C9, asymmetric units of (I) and its sister compound [II; Liu *et al.* (2010)] show almost identical geometries (see Supplementary material). Bond lengths and angles as well as the C4–C7–N1–C8 torsion angle match well with the reported average values for *p*-R'–C_6_H_4_–CH=N–C_6_H_4_–R"-*p* [analysis of the Cambridge Structural Database (CSD); Version 5.27, release February 2009; Allen, 2002; 128 entries, 173 fragments]. Fragments O1/C1—C7/N1/C18 and C8—C13/N2/C14/C16 are almost planar [within 0.09 and 0.06 Å]. The amino N2 atom is also in a planar environment [sum of the valent angles 359.9 (3)°] which most frequenty the case for aryldialkylamines (range from 317.6 to 360.0°, average value 359.0°).

In (I), both hydroxy H-atoms are involved into hydrogen bonding [for the H-bonds lengths and angles values, see the Table]. In (I), molecules along with their equivalents generated by a 2_1_ screw axis form a one-dimensional infinite chain stretched along the *b*-axis. Organic moieties are oriented outwards the corresponding screw axis (see Fig. 2). These one-dimensional assemblies do not interact with their equivalent neighbours by any hydrogen bonds and are just stacked one by another. This results in an evident flattening of the entire CH_3_O—C_6_H_4_C(H)=NC_6_H_4_ moieties for the inter-chain repulsion diminishing. Crystal lattice packing of (I) differs markedly from that of (II) (Liu *et al.*, 2010)).

### Experimental

1-Chloro-4-nitrobenzene, 4-methoxy-benzaldehyde, 2-(2-hydroxy-ethylamino)-ethanol, ammonium formate, 10% Pd/C catalyst and solvents were purchased from Sinopharm Chemical Reagent and Tianjin Fuyu Chemical companies. 2-[(2-Hydroxy-ethyl)-(4-nitro-phenyl)-amino]-ethanol was prepared as described by Cho & Park (1997) and Ferlin *et al.* (2004). Reduction of the nitro-group was carried out as described by Lewis *et al.* (2009). Schiff-base preparation was done by a modification of the procedure reported by von König *et al.* (1982).

Procedure: 1-chloro-4-nitrobenzene (15.76 g, 0.10 mol) was dissolved in 2-(2-hydroxy-ethylamino)-ethanol (50 ml). The reaction mixture was heated to 393 K for 10 h and then cooled down to room temperature. Precipitating crude 2-[(2-Hydroxy-ethyl)-(4-nitro-phenyl)-amino]-ethanol was filtered off, dried in vacuum and recrystallized from a minimal amount of hot ethanol. Yield 11.54 g (51%). 2-[(2-Hydroxy-ethyl)-(4-nitro-phenyl)-amino]-ethanol (8.15 g, 0.036 mol) was then dissolved in MeOH (50 ml). To this solution, HCOONH_4 _(0.216 mol) and 10% Pd/C (0.6 g) were added and the slurry was stirred at 293 K for 30 min. On removal of the catalyst by filtration, the filtrate was placed into a N_2_-flushed flask containing 1 ml of acetic acid and an equimolar (0.036 mol) amount of 4-methoxybenzaldehyde (0.036 mol) was added dropwise at 333 K during 30 min. The reaction mixture was kept at the same temperature for additional 30 min, cooled down to 273 K and ice-cold water (200 ml) was added. The precipitated yellow solid was collected by filtration, washed with water, dried under reduced pressure and, finally, re-crystallized by a slow evaporation of its methanolic solution in air at 293 K. Yield 95%, m.p. 402 K. ^1^H NMR (I) δ: 8.50 (s, 1 H, CH═N), 6.78–7.83 (m, 8 H, C_6_H_4_), 3.31, 3.72 (both t, 4 H and 4 H, ^3^*J*_HH_ = 7.2 Hz, CH_2_), 3.86 (s, 3H, CH_3_). A ingle crystal of (I) suitable for X-ray diffraction analysis was picked up directly from the obtained material.

### Refinement

All non-H atoms were refined anisotropically. H atoms except of H7 and OH were treated as riding atoms with distances of C—H = 0.96 (CH_3_), 0.97 (CH_2_), 0.93 Å (C_Ar_H), and *U*_iso_(H) = 1.5 *U*_eq_(C), 1.2 *U*_eq_(C), and 1.2 *U*_eq_(C), respectively. Atoms H7 and OH hydrogen atoms were found from difference Fourier syntheses and refined isotropically. Despite the fact that an achiral compound (I) crystallizes in a chiral space group *P*2_1_, neither the absolute structure determination nor approval of the inversion twinning was possible due to evident reasons (Mo-*K*α radiation with no atoms heavier than oxygen) and the refinement for (I) was preformed with the Friedel opposites merged (MERG 3 instruction).

### Crystal data

**Table:**

C_18_H_22_N_2_O_3_	*F*(000) = 336
*M**_r_* = 314.38	*D*_x_ = 1.300 Mg m^−^^3^
Monoclinic, *P*2_1_	Mo *K*α radiation, λ = 0.71073 Å
Hall symbol: P 2yb	Cell parameters from 2324 reflections
*a* = 5.3795 (9) Å	θ = 2.0–28.2°
*b* = 8.0585 (14) Å	µ = 0.09 mm^−^^1^
*c* = 18.531 (3) Å	*T* = 296 K
β = 91.168 (2)°	Block, yellow
*V* = 803.2 (2) Å^3^	0.24 × 0.13 × 0.07 mm
*Z* = 2	

### Data collection

**Table:**

Bruker SMART APEXII diffractometer	1541 independent reflections
Radiation source: fine-focus sealed tube	1225 reflections with *I* > 2σ(*I*)
graphite	*R*_int_ = 0.037
Detector resolution: 8.333 pixels mm^-1^	θ_max_ = 25.1°, θ_min_ = 2.2°
phi and ω scans	*h* = −5→6
Absorption correction: multi-scan (*TWINABS*; Sheldrick, 1996)	*k* = −9→9
*T*_min_ = 0.979, *T*_max_ = 0.994	*l* = −22→20
4036 measured reflections	

### Refinement

**Table:**

Refinement on *F*^2^	Secondary atom site location: difference Fourier map
Least-squares matrix: full	Hydrogen site location: inferred from neighbouring sites
*R*[*F*^2^ > 2σ(*F*^2^)] = 0.038	H atoms treated by a mixture of independent and constrained refinement
*wR*(*F*^2^) = 0.108	*w* = 1/[σ^2^(*F*_o_^2^) + (0.0702*P*)^2^] where *P* = (*F*_o_^2^ + 2*F*_c_^2^)/3'
*S* = 1.06	(Δ/σ)_max_ < 0.001
1541 reflections	Δρ_max_ = 0.17 e Å^−^^3^
222 parameters	Δρ_min_ = −0.13 e Å^−^^3^
1 restraint	Extinction correction: *SHELXL97* (Sheldrick, 2008), Fc^*^=kFc[1+0.001xFc^2^λ^3^/sin(2θ)]^-1/4^
Primary atom site location: structure-invariant direct methods	Extinction coefficient: 0.028 (7)

### Special details

**Table:**

Geometry. All esds (except the esd in the dihedral angle between two l.s. planes) are estimated using the full covariance matrix. The cell esds are taken into account individually in the estimation of esds in distances, angles and torsion angles; correlations between esds in cell parameters are only used when they are defined by crystal symmetry. An approximate (isotropic) treatment of cell esds is used for estimating esds involving l.s. planes.
Refinement. Refinement of *F*^2^ against ALL reflections. The weighted *R*-factor wR and goodness of fit *S* are based on *F*^2^, conventional *R*-factors *R* are based on *F*, with *F* set to zero for negative *F*^2^. The threshold expression of *F*^2^ > 2σ(*F*^2^) is used only for calculating *R*-factors(gt) etc. and is not relevant to the choice of reflections for refinement. *R*-factors based on *F*^2^ are statistically about twice as large as those based on *F*, and *R*- factors based on ALL data will be even larger.

### Fractional atomic coordinates and isotropic or equivalent isotropic displacement parameters (Å^2^)

**Table:**

	*x*	*y*	*z*	*U*_iso_*/*U*_eq_	
O1	0.5558 (4)	0.2396 (3)	−0.24985 (10)	0.0589 (6)	
O2	−0.3959 (5)	−0.0877 (3)	0.43576 (14)	0.0712 (8)	
O3	−0.4849 (5)	0.5749 (3)	0.43870 (13)	0.0628 (7)	
N1	0.1089 (5)	0.2496 (3)	0.06704 (12)	0.0469 (6)	
N2	−0.1774 (5)	0.2079 (3)	0.35822 (11)	0.0515 (7)	
C1	0.5101 (5)	0.2264 (4)	−0.17743 (13)	0.0424 (7)	
C2	0.6533 (6)	0.1361 (4)	−0.12960 (15)	0.0485 (7)	
H2A	0.7947	0.0811	−0.1450	0.058*	
C3	0.5840 (6)	0.1278 (4)	−0.05799 (15)	0.0476 (7)	
H3A	0.6830	0.0680	−0.0255	0.057*	
C4	0.3737 (6)	0.2052 (4)	−0.03335 (14)	0.0427 (7)	
C5	0.2341 (5)	0.3012 (4)	−0.08283 (14)	0.0466 (7)	
H5	0.0939	0.3577	−0.0675	0.056*	
C6	0.3029 (6)	0.3123 (4)	−0.15342 (15)	0.0487 (8)	
H6	0.2106	0.3776	−0.1855	0.058*	
C7	0.3019 (6)	0.1862 (4)	0.04166 (16)	0.0494 (8)	
C8	0.0474 (5)	0.2305 (4)	0.14073 (14)	0.0416 (7)	
C9	0.1730 (6)	0.1309 (4)	0.19122 (15)	0.0471 (7)	
H9	0.3095	0.0692	0.1769	0.057*	
C10	0.0986 (5)	0.1223 (4)	0.26191 (15)	0.0434 (7)	
H10	0.1849	0.0537	0.2940	0.052*	
C11	−0.1033 (5)	0.2140 (3)	0.28648 (14)	0.0405 (7)	
C12	−0.2278 (5)	0.3138 (4)	0.23556 (14)	0.0474 (7)	
H12	−0.3625	0.3774	0.2498	0.057*	
C13	−0.1543 (5)	0.3197 (4)	0.16480 (14)	0.0478 (7)	
H13	−0.2432	0.3857	0.1322	0.057*	
C14	−0.0579 (6)	0.0964 (4)	0.40875 (15)	0.0508 (8)	
H14B	−0.0800	0.1388	0.4572	0.061*	
H14A	0.1190	0.0952	0.3997	0.061*	
C15	−0.1541 (6)	−0.0790 (4)	0.40534 (16)	0.0562 (8)	
H15A	−0.1624	−0.1158	0.3555	0.067*	
H15B	−0.0413	−0.1519	0.4318	0.067*	
C16	−0.3805 (6)	0.3088 (4)	0.38402 (15)	0.0488 (8)	
H16B	−0.4516	0.2560	0.4259	0.059*	
H16A	−0.5092	0.3158	0.3467	0.059*	
C17	−0.2951 (6)	0.4822 (4)	0.40415 (17)	0.0547 (8)	
H17B	−0.1508	0.4747	0.4362	0.066*	
H17A	−0.2457	0.5403	0.3609	0.066*	
C18	0.7676 (6)	0.1549 (5)	−0.27577 (17)	0.0647 (10)	
H18C	0.7739	0.1667	−0.3273	0.097*	
H18A	0.7567	0.0394	−0.2636	0.097*	
H18B	0.9154	0.2013	−0.2540	0.097*	
H2	−0.428 (9)	−0.200 (7)	0.438 (2)	0.116 (18)*	
H3	−0.542 (8)	0.522 (6)	0.473 (2)	0.094 (16)*	
H7	0.417 (7)	0.122 (5)	0.072 (2)	0.070 (10)*	

### Atomic displacement parameters (Å^2^)

**Table:**

	*U*^11^	*U*^22^	*U*^33^	*U*^12^	*U*^13^	*U*^23^
O1	0.0670 (15)	0.0725 (15)	0.0376 (10)	0.0002 (13)	0.0076 (10)	0.0024 (11)
O2	0.0969 (19)	0.0440 (14)	0.0745 (16)	−0.0085 (13)	0.0465 (14)	−0.0068 (12)
O3	0.1009 (19)	0.0386 (12)	0.0500 (13)	0.0089 (12)	0.0287 (12)	0.0006 (11)
N1	0.0500 (15)	0.0515 (15)	0.0395 (12)	0.0011 (13)	0.0089 (11)	−0.0011 (12)
N2	0.0741 (18)	0.0424 (14)	0.0388 (13)	0.0072 (14)	0.0176 (12)	0.0014 (11)
C1	0.0462 (17)	0.0451 (16)	0.0362 (14)	−0.0077 (15)	0.0069 (12)	−0.0046 (14)
C2	0.0472 (17)	0.0520 (18)	0.0469 (16)	0.0042 (15)	0.0118 (13)	−0.0010 (15)
C3	0.0505 (17)	0.0494 (18)	0.0431 (16)	0.0040 (15)	0.0041 (13)	0.0035 (14)
C4	0.0463 (17)	0.0440 (18)	0.0380 (14)	−0.0032 (14)	0.0044 (12)	−0.0022 (13)
C5	0.0446 (16)	0.0497 (18)	0.0454 (16)	0.0039 (15)	0.0030 (13)	−0.0061 (14)
C6	0.0501 (17)	0.0525 (18)	0.0431 (15)	0.0013 (16)	−0.0041 (13)	0.0026 (15)
C7	0.055 (2)	0.053 (2)	0.0408 (16)	0.0006 (17)	0.0054 (15)	−0.0003 (14)
C8	0.0459 (16)	0.0408 (16)	0.0384 (14)	−0.0046 (14)	0.0062 (12)	−0.0015 (14)
C9	0.0505 (17)	0.0436 (17)	0.0477 (16)	0.0027 (15)	0.0124 (13)	−0.0021 (14)
C10	0.0499 (17)	0.0405 (16)	0.0400 (15)	0.0022 (14)	0.0063 (13)	0.0026 (13)
C11	0.0524 (18)	0.0319 (15)	0.0375 (14)	−0.0050 (13)	0.0077 (12)	−0.0007 (12)
C12	0.0472 (17)	0.0478 (17)	0.0477 (16)	0.0060 (15)	0.0129 (13)	−0.0021 (15)
C13	0.0499 (18)	0.0497 (17)	0.0439 (16)	0.0055 (16)	0.0029 (13)	0.0042 (15)
C14	0.0660 (19)	0.0506 (19)	0.0360 (14)	−0.0014 (16)	0.0083 (13)	0.0003 (14)
C15	0.074 (2)	0.0450 (18)	0.0506 (17)	0.0049 (17)	0.0215 (15)	0.0067 (15)
C16	0.064 (2)	0.0413 (17)	0.0423 (15)	−0.0047 (15)	0.0172 (14)	−0.0018 (14)
C17	0.074 (2)	0.0420 (17)	0.0483 (16)	−0.0061 (16)	0.0156 (15)	−0.0042 (14)
C18	0.063 (2)	0.084 (3)	0.0475 (18)	−0.010 (2)	0.0156 (16)	−0.0099 (18)

### Geometric parameters (Å, °)

**Table:**

O1—C1	1.373 (3)	C8—C13	1.383 (4)
O1—C18	1.420 (4)	C8—C9	1.396 (4)
O2—C15	1.430 (4)	C9—C10	1.379 (4)
O2—H2	0.92 (5)	C9—H9	0.9300
O3—C17	1.427 (4)	C10—C11	1.398 (4)
O3—H3	0.82 (5)	C10—H10	0.9300
N1—C7	1.257 (4)	C11—C12	1.400 (4)
N1—C8	1.420 (3)	C12—C13	1.378 (4)
N2—C11	1.397 (3)	C12—H12	0.9300
N2—C14	1.440 (4)	C13—H13	0.9300
N2—C16	1.451 (4)	C14—C15	1.506 (5)
C1—C2	1.372 (4)	C14—H14B	0.9700
C1—C6	1.393 (4)	C14—H14A	0.9700
C2—C3	1.387 (4)	C15—H15A	0.9700
C2—H2A	0.9300	C15—H15B	0.9700
C3—C4	1.378 (4)	C16—C17	1.515 (4)
C3—H3A	0.9300	C16—H16B	0.9700
C4—C5	1.405 (4)	C16—H16A	0.9700
C4—C7	1.458 (4)	C17—H17B	0.9700
C5—C6	1.369 (4)	C17—H17A	0.9700
C5—H5	0.9300	C18—H18C	0.9600
C6—H6	0.9300	C18—H18A	0.9600
C7—H7	0.98 (4)	C18—H18B	0.9600
			
C1—O1—C18	117.0 (2)	N2—C11—C12	121.3 (2)
C15—O2—H2	104 (3)	C10—C11—C12	116.6 (2)
C17—O3—H3	111 (3)	C13—C12—C11	121.2 (3)
C7—N1—C8	121.7 (3)	C13—C12—H12	119.4
C11—N2—C14	120.6 (2)	C11—C12—H12	119.4
C11—N2—C16	121.7 (2)	C12—C13—C8	122.1 (3)
C14—N2—C16	117.7 (2)	C12—C13—H13	119.0
C2—C1—O1	124.3 (3)	C8—C13—H13	119.0
C2—C1—C6	120.0 (2)	N2—C14—C15	114.2 (3)
O1—C1—C6	115.7 (2)	N2—C14—H14B	108.7
C1—C2—C3	119.0 (3)	C15—C14—H14B	108.7
C1—C2—H2A	120.5	N2—C14—H14A	108.7
C3—C2—H2A	120.5	C15—C14—H14A	108.7
C4—C3—C2	122.2 (3)	H14B—C14—H14A	107.6
C4—C3—H3A	118.9	O2—C15—C14	110.1 (3)
C2—C3—H3A	118.9	O2—C15—H15A	109.6
C3—C4—C5	117.7 (2)	C14—C15—H15A	109.6
C3—C4—C7	120.2 (3)	O2—C15—H15B	109.6
C5—C4—C7	122.1 (3)	C14—C15—H15B	109.6
C6—C5—C4	120.5 (3)	H15A—C15—H15B	108.1
C6—C5—H5	119.7	N2—C16—C17	111.8 (3)
C4—C5—H5	119.7	N2—C16—H16B	109.3
C5—C6—C1	120.4 (3)	C17—C16—H16B	109.3
C5—C6—H6	119.8	N2—C16—H16A	109.3
C1—C6—H6	119.8	C17—C16—H16A	109.3
N1—C7—C4	123.4 (3)	H16B—C16—H16A	107.9
N1—C7—H7	121 (2)	O3—C17—C16	112.2 (3)
C4—C7—H7	115 (2)	O3—C17—H17B	109.2
C13—C8—C9	117.0 (2)	C16—C17—H17B	109.2
C13—C8—N1	117.0 (3)	O3—C17—H17A	109.2
C9—C8—N1	126.0 (3)	C16—C17—H17A	109.2
C10—C9—C8	121.3 (3)	H17B—C17—H17A	107.9
C10—C9—H9	119.4	O1—C18—H18C	109.5
C8—C9—H9	119.4	O1—C18—H18A	109.5
C9—C10—C11	121.7 (3)	H18C—C18—H18A	109.5
C9—C10—H10	119.2	O1—C18—H18B	109.5
C11—C10—H10	119.2	H18C—C18—H18B	109.5
N2—C11—C10	122.0 (3)	H18A—C18—H18B	109.5
			
C18—O1—C1—C2	−0.8 (4)	C8—C9—C10—C11	−0.8 (5)
C18—O1—C1—C6	179.3 (3)	C14—N2—C11—C10	−4.5 (4)
O1—C1—C2—C3	−178.0 (3)	C16—N2—C11—C10	177.3 (3)
C6—C1—C2—C3	1.9 (5)	C14—N2—C11—C12	175.9 (3)
C1—C2—C3—C4	1.1 (5)	C16—N2—C11—C12	−2.3 (4)
C2—C3—C4—C5	−3.0 (5)	C9—C10—C11—N2	−179.0 (3)
C2—C3—C4—C7	177.0 (3)	C9—C10—C11—C12	0.6 (4)
C3—C4—C5—C6	1.9 (4)	N2—C11—C12—C13	−180.0 (3)
C7—C4—C5—C6	−178.0 (3)	C10—C11—C12—C13	0.4 (4)
C4—C5—C6—C1	1.0 (4)	C11—C12—C13—C8	−1.2 (5)
C2—C1—C6—C5	−3.0 (5)	C9—C8—C13—C12	1.0 (4)
O1—C1—C6—C5	177.0 (3)	N1—C8—C13—C12	−178.5 (3)
C8—N1—C7—C4	−179.0 (3)	C11—N2—C14—C15	−81.9 (3)
C3—C4—C7—N1	−178.0 (3)	C16—N2—C14—C15	96.3 (3)
C5—C4—C7—N1	1.9 (5)	N2—C14—C15—O2	−72.9 (3)
C7—N1—C8—C13	173.3 (3)	C11—N2—C16—C17	−83.9 (3)
C7—N1—C8—C9	−6.2 (5)	C14—N2—C16—C17	97.9 (3)
C13—C8—C9—C10	0.0 (4)	N2—C16—C17—O3	−172.0 (2)
N1—C8—C9—C10	179.5 (3)		

### Hydrogen-bond geometry (Å, °)

**Table:**

*D*—H···*A*	*D*—H	H···*A*	*D*···*A*	*D*—H···*A*
O2—H2···O3^i^	0.95 (5)	1.84 (6)	2.762 (3)	177 (4)
O3—H3···O2^ii^	0.82 (4)	1.95 (4)	2.757 (4)	166 (4)

Symmetry codes: (i) *x*, *y*−1, *z*; (ii) −*x*−1, *y*+1/2, −*z*+1.
